# Impact of environmental interventions based on social programs on physical activity levels: A systematic review

**DOI:** 10.3389/fpubh.2023.1095146

**Published:** 2023-03-23

**Authors:** Edgar D. Hernández, Elisa A. Cobo, Lawrence P. Cahalin, Pamela Seron

**Affiliations:** ^1^Facultad de Medicina, Human Movement Department, Universidad Nacional de Colombia, Bogotá, Colombia; ^2^Facultad de Ciencias de la Salud, Universidad de Boyacá, Tunja, Colombia; ^3^Department of Physical Therapy, University of Miami, Coral Gables, FL, United States; ^4^Facultad de Medicina, Universidad de La Frontera, Temuco, Chile

**Keywords:** physical activity, environmental, build environment, natural experiment, programs

## Abstract

**Background:**

The design of social programs at the environmental level such as in schools, parks, bicycle paths, or workspaces generates changes in the behavior of individuals and modifies lifestyles by increasing physical activity (PA) levels.

**Objective:**

To determine the effectiveness of environmental interventions based on social programs by changing the population's level of PA.

**Methodology:**

Natural experiment studies that involved environmental intervention programs at a social level were included. The primary outcome was PA levels with consideration of both objective and subjective measurements. An electronic search was carried out in Medline/Pubmed, SCIENCE DIRECT, WEB OF SCIENCE, and CINAHL databases up to January 2022 with two reviewers screening titles and abstracts and selecting studies for full-text reading. Two reviewers also acquired relevant data and evaluated study quality using the ROBINS I tool. A qualitative analysis was performed.

**Results:**

Three thousand eight hundred and sixty-five articles were found in the 4 consulted databases. After eliminating duplication (200), two reviewers screened 3,665 titles and abstracts and excluded 3,566 that did not meet the inclusion criteria, leaving 99 articles to be read in full text. The 99 full texts were reviewed of which 24 papers met the eligibility criteria. All were natural experiments published between 2011 and 2020 and all evaluated environmental social programs revealing that social programs at the environmental level promoted PA in various populations at the community level worldwide.

**Conclusion:**

The 24 reviewed studies suggest innovative proposals for social programs that seek to increase PA and promote healthy lifestyles related to public activity policies developed in the countries in which they were generated. Environmental social programs can positively impact PA levels among children and adults.

**Systematic review registration:**

https://www.crd.york.ac.uk/prospero/display_record.php?RecordID=229718, identifier: CRD42021229718.

## Introduction

A variety of factors worldwide have altered patterns of physical activity (PA), with an increase in the level of sedentary lifestyles especially in middle and low-income countries (1). Low level of PA is one of the key factors related to the development of chronic diseases. In addition, morbimortality due to chronic diseases secondary to physical inactivity has been found to be related to the worldwide prevalence of type 2 diabetes, coronary artery disease, and cancer among others, with relative risk (RR) of physical inactivity of 1.16 (95% CI 1.04–1.30)for all causes worldwide (2).

The world's economic and health behavior changes, such as an increase in working hours especially for mothers, unhealthy food consumption, a reduction in leisure and recreation time, the reduced metabolic expenditure given the influence of the obesogenic space because of less PA and the imbalance between intake and consumption lead to the presence of childhood and adult obesity (3). In this sense, the evidence shows that the economic market has a substantial influence on the commercial aspect of food in advertising for children, television time, and high investments in fast food restaurants. In contrast to the above investments there is a low investment in environmental modifications that may have a greater effect on the time spent in PA which may have a favorable effect on the imbalance between intake and demand and decrease obesity (4, 5).

Health policies, especially the guidelines generated by the World Health Organization (WHO) are aimed to increase PA in populations, with a minimum of 150 min and nutritional improvement with a balanced diet, to reduce the presence of chronic non-communicable diseases (6, 7). Moreover, PA interventions or strategies at the individual, community, environmental, and social levels may favorably alter poor health behaviors and have a positive impact on levels of PA improving the population's general health and chronic non-communicable diseases (8, 9).

The WHO describes environmental strategies regarding social programs as global actions, community approaches and public policy developed to allow the implementation of social determinants of health within community spaces as schools, parks, bicycle routes, bicycle lanes, companies, or cities (10–13). Examples of such programs have been implemented worldwide as methods to promote PA (1, 14, 15). In the United Kingdom and other countries, national guidelines have been developed for environmental modification and the creation of social programs to encourage greater PA and reduce sedentary lifestyles (16, 17). The conceptual background of environmental social programs has been described in international documents such as the National Institute for Health and Care Excellence (NICE) in which the role of PA is highlighted stating “Local strategies, policies and plans to encourage and enable people to be more physically active” (18). The socio-ecological models of PA explain how to facilitate and implement PA at different levels of the individual including behavioral, social, and physical environmental constructs (19, 20). For example, schools with in-school or out-of-school programs for children are likely to promote more PA and less sedentary lifestyles. In addition, the implementation of active walking or cycling routes, modification of cities with the inclusion of active programs, and the reduction of spaces that induce obesity to reduce the level of sedentary lifestyles in men, women, and children throughout the life cycle have been reported (15, 21–25).

Worldwide, environmental modification programs from the social perspective have gained relevance for the implementation of policy-based programs in countries whose impact has been evaluated through natural experiments. Natural experiments have been described as observational studies in which an event or a situation that allows for the random or seemingly random assignment of study subjects to different groups is used to answer a particular question (24). Thus, natural experiments can observe large populations in a real-world environment to examine the effects of global actions or community approaches. The medical research council has recommended the use of natural experimental approaches to evaluate population health interventions. Thus, natural experiments are extremely important since the exposure to an event or intervention of interest has not been manipulated by a researcher making the natural experiment not only an observational study, but an experimental study especially when a clinical trial may be impractical or unethical (26).

The effect of social programs at the environmental level can be assessed by natural experiments and appear to generate favorable change in the behaviors and lifestyles of individuals in work, school, and university settings. The use of transportation methods to facilitate healthy lifestyle habits has also been suggested as a strategy to improve PA, however, the results are inconclusive (15, 27–30). Therefore, this systematic review aims to examine the effectiveness of environmental interventions based on programs at a social level on levels of PA in studies that have employed natural experiments.

## Methodology

This study is a systematic review conducted according to the guidelines of Cochrane methodology (31) and PRISMA guidelines (32). The protocol is registered in the international database of systematic reviews PROSPERO under the number CRD42021229718.

### Selection criteria

#### Type of study

The type of study is natural experiments that involve environmental interventions at the social level which includes local strategies, plans, programs, or policies to promote PA with the understanding that a natural experiment is a research study in which the exposure to an event or intervention of interest has not been manipulated by a researcher (26).

#### Type of participants or population of interest

The type of participants and population of interest includes the general population such as students in schools and universities, individuals in the workplace, the population of individuals in cities and neighborhoods, and older adults in institutions. Studies that have targeted specific populations with diseases or conditions such as neuromuscular disease (sclerosis, cerebrovascular disease, and dystrophies), musculoskeletal diseases (lupus, arthritis, and osteoarthritis), or cardiovascular diseases (infarction, arrhythmia, valve diseases, etc.) as well as studies on athletes were excluded.

#### Type of interventions

Studies that evaluated programs focused on the promotion of PA from an environmental perspective at the social level such as programs involving parks, bicycle commuting, bicycle lanes, school curriculum modifications, or city programs to promote PA such as muevete and recreovia Bogota, Biking Barcelona, Biking Boulevards Australia, Agita São Paulo, role of public policy in active schools in Ontario, and others.

#### Type of outcomes

The primary outcome is PA defined as variation or levels reached and measured objectively or subjectively. Objective measurements included PA measured by pedometers, accelerometers, heart rate monitors, and direct and indirect calorimetry. Subjective measurements included self-reports or questionnaires such as the IPAQ, CHAMPS, or the PA Recall, among others. Both the objective and subjective measurements could be expressed continuously [such as total energy expenditure (Kcal/Kg/week, kcal/week), metabolic consumption in METS, oxygen consumption or differences in CO_2_/Vo_2_, heart rate, heart variability, total minutes of physical activity or the number of steps, among others] or categorically (such as of light, moderate, or vigorous PA). Participation in the programs, percentage or amount of PA performed, measures of fitness level if they were reported in metabolic expenditure or oxygen consumption, and measurement scales of individual or group physical activity were also examined.

### Search strategy

A search for studies was performed through January 2022 using the following electronic databases: Medline/Pubmed, Web of Science, Science Direct, and CINAHL, using Mesh, Decs, and Emtree terms. Appendix 1 provides the search strategies employed in the study. Additionally, a search of crossed references was done manually as well as a search of gray literature in specialist journals, university repositories or general websites related to the topic.

### Study selection

Study selection involved two reviewers (EH, EC) who screened the articles by title and abstract according to the inclusion criteria. The selected studies were then blinded, read in full text format by both reviewers and the results and conclusions compared. In the case of disagreements, a third reviewer acted as a peer evaluator to settle disagreements for the definitive selection of studies. To optimize the work at this stage, the Rayyan^©^ software was used (33).

### Data extraction and risk of bias evaluation

Data extraction was accomplished using a spreadsheet in which the characteristics of the studies were recorded, such as title, authors, year and place of publication, program undertaken and its characteristics, start and end date, the scope of the program's intervention, outcomes and considered measurements, and reported results.

The risk of bias was assessed using the recommendations and evaluation criteria of the ROBINS I tool for non-randomized intervention studies (34). Which made it possible to evaluate specific risks of bias at three points in the study: (1) pre-intervention where the bias of confounding and participant selection were considered, (2) during the intervention where measurement bias was assessed, and (3) post-intervention where the bias of the interventions performed and outcome measurements as well as attrition bias were considered.

### Data analysis

The data analysis was undertaken qualitatively through figures and tables showing the information obtained in the data extraction process which provided an organized and visual presentation of the intervention programs, methodological findings, and results of each study.

## Results

**Figure 1:**
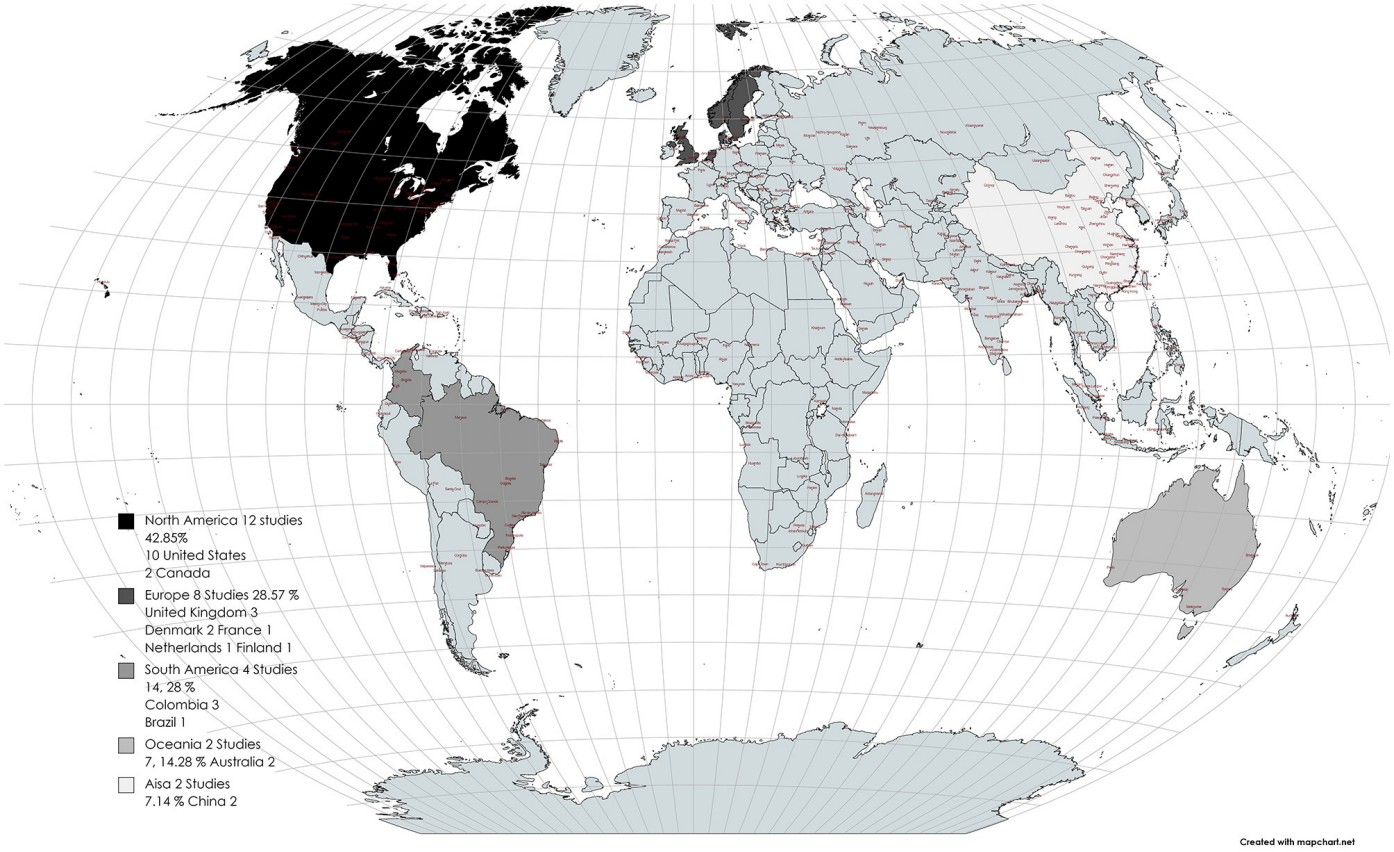
Study distribution around the world.

Three thousand eight hundred and sixty-five articles were found in the 4 databases. After eliminating duplicates (200), two reviewers screened 3,665 titles and abstracts and excluded 3,566 articles because they did not meet the inclusion criteria. Thus, 99 full texts were reviewed in depth to determine that 28 studies fulfilled the eligibility criteria. The reasons for exclusion were: 20 articles were not natural experiments, 27 did not have outcomes of interest, and 24 were natural experiments but focused on physical environmental structural modification. It is important to note that of these 28 full-text readings, 4 had double reporting in different articles, resulting in a total of 24 reports. Of these 24, 7 studies examined both physical environmental modifications and social programs. The 24 papers used in this study as well as those with double reports are shown in Figure 1.

### Characteristics of the selected studies

**Figure 2:**
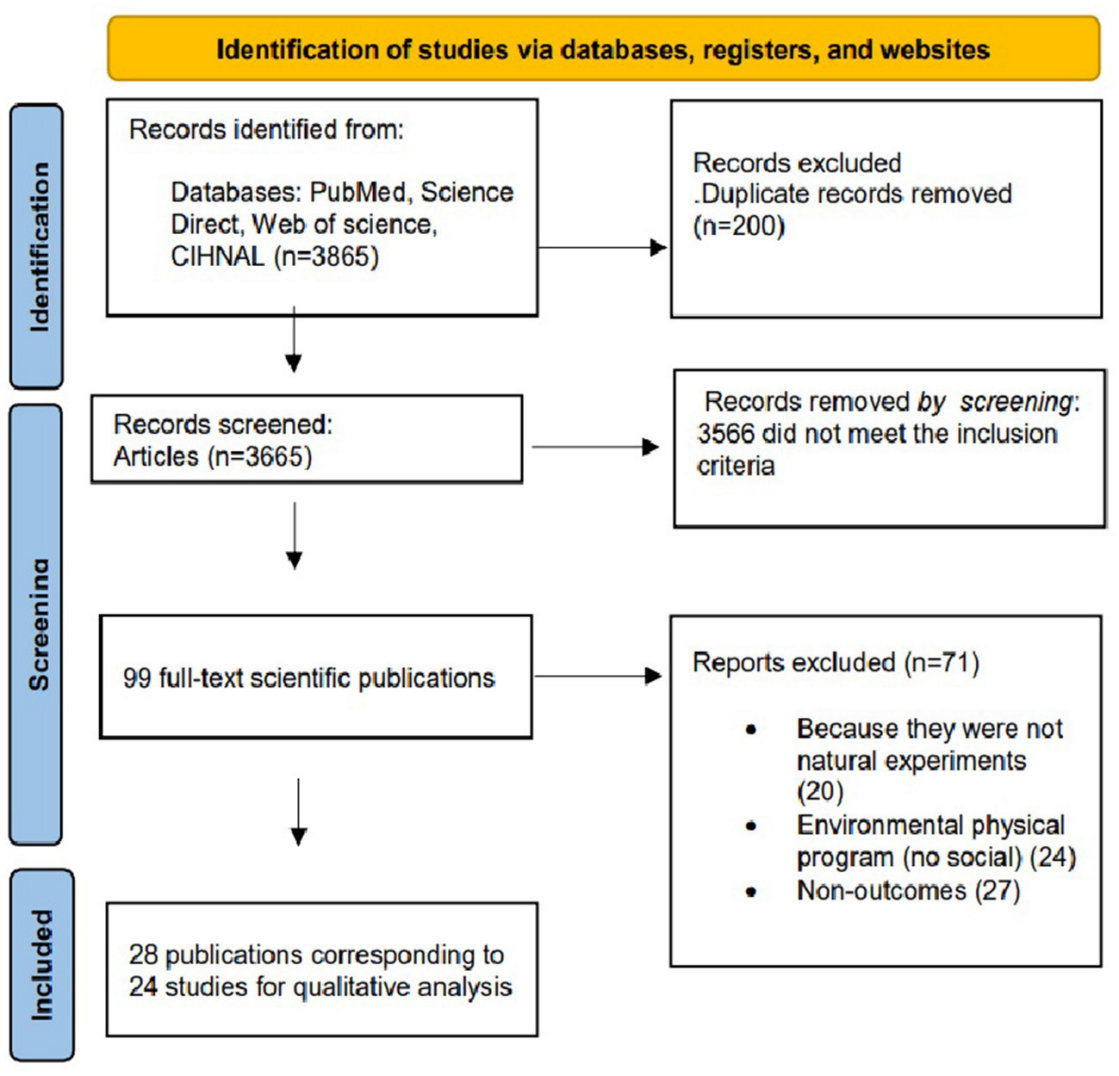
Flowchart of the selection process.

The 28 study reports (35–62). That were selected were natural experiments published between 2011 and 2020, with the evaluation of environmental social programs from different parts of the world (Figure 2). Regarding the scope, 10 were implemented at school programs, 6 were related to active transportation, 4 were in active cities, 2 were in parks, and 2 were in workspaces (Table 1).

**Table 1:** Characteristics of the studies included and areas of emphasis.

**References**	**Country/Continent**	**Infrastructural focus**	**Intervention program**	**Description of the group**
Dill et al. (36)	United States/North America	Active transport	Bicycle paths were planned and built and compared to the control.	Six studies focus on the implementation of active transportation or commutation, whether understood as walking spaces, bicycle programs, or transportation interchange between bus and walking. Four of these studies focus on bicycle programs in the community environment to encourage the use of this form of transportation, focusing on commuting to work. And two of the subway system in which the use of non-escalators vs. escalators at station exits is encouraged with a cognitive program to encourage the use of escalators and the other on a new subway line measures the use of commutation transportation related to transportation to the subway line and distance to stations with the objective of encouraging physical activity.
Panter et al. (37)	United Kingdom/Europe	Active transport commute	A new transportation system was built in Cambridge between 2007 and 2011 19 kilometers of busway and bike/walking lanes and the commuter program was implemented.	
Goodman et al. (40)	United Kingdom/Europe	Active transport bicycling	18 cities with bicycle programs were included for use of bicycles for commuting to work, school, bus and subway stations.	
Allais et al. (46)	France/Europe	Active transport Activity promotion	worked with three stations of the French subway to encourage the use of stairs, three experimental and control stations to see the change in the pattern of use of stairs.	
Sun et al. (51)	China/Asia	Active transport	A new metro line with 24 stations in a population that had no metro line and the change of habits in the type of transport was determined.	
Heinen et al. (60)	Australia/Oceania	Active transport	Based on the habitat cohort in which a program is designed in which we seek to look at changes in the pattern of transport with cycling and its effect on physical activity	
Simões et al. (41)	Brazil /South America	Active community cities	Academy program of city of Pernambuco Brazil with 184 cities that participated in the activity in three groups.	4 studies carried out in urban environments at the community level, all different from each other but with the same perspective, which is the community work to promote physical activity, within the framework of the activity policy.
Nicosia and Datar (50)	United States/North America	Active community cities	Projected exercise and nutrition environment of military housing, measured days of physical activity with activity minutes and follow-up.	
Mölenberg et al. (52)	Holland/Europe	Active community cities	18 new spaces in economically depressed sectors, in which spaces for the promotion of physical activity were created in Rotterdam.	
Sharma et al. (54)	United States/North America	Active community cities	Multicomponent healthy eating and physical activity program for pregnant women, the program promotes breastfeeding, nutrition and physical activity, community-based 6-week program	
Gesell et al. (38)	United States/North America	After School Program	After-school activity program with the department parks with its modified areas and spaces for physical activity at the community level.	10 studies focused on educational environments, 9 in schools and 1 at the university level, all based on environmental social activities either inside or outside the educational environment but with the objective of promoting physical activity. Of the 10 school-based, 6 are associated with the curriculum and 4 are after school associated with summer programs or park activities. The program at the university level is focused on determining whether the distance between university dormitories to gyms and dining halls influences the level of physical activity of students.
Esdaile et al. (42)	Australia/Oceania	Intra-school School Program	Parent-led physical activity and nutrition program known as PEACH, which consists of a 90-min activity program for school children.	
Hunt et al. (44)	United States/North America	After-school program	First and second grade students were included in a 7-week summer learning and activity program.	
Lee et al. (45)	United States/North America	School infrastructure	Transfer of a group of students at a school outside a neighborhood to one with the school in the neighborhood and to see the change in behavior from sedentary to active and to the transport.	
Tarp et al. (48)	Denmark/Europe	Interschool school program	Effect of the multimodal CHAMPS (Childhood Health, Activity, and Motor Performance School study DK) program in an intervention group relative to the control group in response to presenting risk factors in 10 schools in Denmark.	
D'Agostino et al. (49)	United States/North America	After School Program	A 10-month after-school program called FIT2PLAY, generated for ethnic minorities in a Miami county.	
Kapinos et al. (53)	United States/North America	College program	To determine how proximity of dormitories to the gymnasium or dining areas or food courts influences freshmen college students' weight gain or sedentary behavior and obesogenic environment	
Madsen (55)	United States/North America	After school program	California fitness program evaluates the physical condition of children in grades 5–7th and 9th through the Fitness Gram.	
Stone et al. (58)	Canada /North America	In-school school program	The study takes place in Ontario's statewide policy of a minimum of 20 min per day of moderate and vigorous physical activity in school structure and schedules, 16 districts are involved	
Azevedo et al. (59)	United Kingdom/Europe	In-school school program	Dance mat program for students in grades 9–11 at the school level to see the effect on physical activity level, 16 mats and a driving unit were delivered to the schools to be used for 12 weeks.	
Torres et al. (35)	Colombia/Sur América	Parks and surrounding recreovia	Effect of physical activity in free time on participants in 9 parks in the capital district of Bogotá	Two programs focused on parks aimed at promoting physical activity with the development of physical activity classes in the free time in the participants in recreational areas, one uses the frozen roads to create trails for walking, measures the use of these and the activity physical activity developed in contrast the other works with the development of physical activity classes in the city and how this allows to modify the life habits of the populations
McGavock et al. (62)	Canada /North America	Parks and surrounding	Impact of a frozen pathway on the users' visits to estimate activity patterns Physical activity associated with the pathway in the winter in Canada.	
Zhu et al. (47)	China/Asia	Work environment	PA and sedentary behavior among the employees of a company in response to job modification, it is a two-arm experiment, one of intervention with modification of the job, adaptation of the chairs and desks.	2 experiments, one from the Asian region and the other from Europe, both focus on physical activity at work, with adaptation of the spaces and the inclusion of adjustable desks and furniture that allow the practice of activity, or the switching of the type of transportation of the company to homes with adaptation of lanes and roads for the use of bicycles and walking.
Aittasalo et al. (57)	Finland/Europe	Work environment active transport	Two-arm natural experiment with two groups of companies in a two-phase socioecological model to determine the use of the bicycle or walking as a means of active transport.	

### Designs of the natural experiments found

Several approaches were used in the natural experiments as shown in Table 2. From pre- and post-cohorts with a control group or prospective cohorts (36, 38, 43, 52, 53, 60), controlled before and after studies (40, 46–48, 57, 59, 62) before and after studies with and without a control group (49–51, 54, 55), as well as quasi-experimental (37, 56), cross-sectional pre- and post-intervention studies with or without a control group (35, 39, 41, 42, 58, 61) providing repeated measures and retrospective studies (44, 45). The 24 studies indicated that they were working on PA policies worldwide or nationally, but 25% did not specify the specific PA policy Using natural experiments to evaluate population health interventions: new Medical Research Council guidance (36, 44, 47, 51, 59, 62), 37.5% described the PA policy, but did not evaluate its development (35, 37, 40–42, 55, 57, 58, 60) and the remaining studies were framed within national public policies on PA or active transportation clearly showing the evaluation of implementation at the level of parks, cities, schools or active transportation (Table 2).

**Table 2:** Main methodological characteristics of the studies.

**References**	**Population**	**Groups**	**Physical activity measurements**	**Type of natural experiment**	**Public policy**
Torres et al. (35)	1,533 participants over 18 years of age in the recreation track and 9 parks	3 groups of parks with new recreation track, old recreation track and without recreation track	IPAQ Accelerometers MVPA Activity level	Cross-sectional Pre-post comparison with control group	Recreovía program in Bogotá, Colombia, Muevete Bogota
Dill et al. (36)	8 bicycle lanes and 11 control streets, 353 adults	Pre- and post-intervention with two groups in bicycle lanes	IPAQ Accelerometers MVPA level of activity Location with GPS	Cohort pre-post with control group	Part of the policy but does not express it,
Prinss et al. (37)	8,783 participants 175 trips 40,000 users	1 km green corridor compared to control group	SOPARC Physical activity level MPA—MVPA	Quasi-experimental nested in a cohort	Be active, be healthy: a plan for getting the nation moving London: Department of Health; 2009.
Gesell et al. (38)	400–800 users in the 4 parks	Two intervention parks and two control parks	Level of physical activity MPA—MVPA Metabolic expenditure in METS	Prospective cohort	Strategic Plan for NIH Obesity Research. Shaping America's Youth. White House Task Force on Childhood Obesity.
Barradas et al. (39)	1,533 participants over 18 years of age in the recreation track and 9 parks	3 groups of parks with new recreation track, old recreation track and without recreation track	IPAQ Accelerometers MVPA Activity level	Cross-sectional Pre-post comparison with control group	Recreovía program in Bogotá, Colombia, Muevete Bogota
Goodman et al. (40)	1,164 individuals, both genders	New transportation system in Cambridge 19 kilometers of busway and bike/walking lanes	RPAQ Accelerometers MVPA Activity level GPS location monitored	Controlled before and after	Cycling-England-cycling-city-and-towns-end-of-program
Simões et al. (41)	8,900 users in 84 cities in Pernambuco	Two intervention groups with modification and physical activity programs and control without modification	IPAQ leisure and transport Level of activity according to walking and participation	Cross-sectional Pre-post nested	Academia da Cidade (AC-R) program of the city of Recife (AC-R), a supervised classes
Esdaile et al. (42)	926 children 816 families	Two groups, one UTC and one TCT each to determine weight loss and activity.	Sociodemographic data Parental data according to economic level and poverty index.	Cross-sectional pre and post with control group	Queensland Health. The Health of Queenslanders 2016. Report of the Chief Health Officer Queensland. Brisbane: Queensland Government
Kapinos and Yakusheva (43)	237 Students assigned to dormitories	Pre- and post-intervention, groups were worked according to distance from the dormitories to gyms and restaurants	Questionnaire Height and weight data, sociodemographic aspects Exercise and diet data with direct questions of frequencies and number	Pre- and post-cohort with control group	U.S. Department of Health and Human Services. Healthy people 2010: With understanding and improving health and objectives for improving health, In: Services USDoHaH, Washington, DC
Hunt et al. (44)	31 children average 6 year old and parents	Pre- and post-intervention to a group with an after school program	Height and weight measures Cardiorespiratory fitness 20-meter running test and PACER Activity measurement with accelerometer Activity management forms for child self-reporting time spent on daily activities such as watching television, home work time, and computer and video games	Repeated measures	Part of the policy but does not express it,
Lee et al. (45)	165 surveys of students were processed	Two intervention groups with modification of a neighborhood school	Survey of forms of transport to and from school in children who were transferred and level of activity	Retrospective	The federal Safe Routes to School (SRTS) programs, pedestrian safety trainings at local schools, Walking School Bus (WSB) programs (a group of students walking to school together led by an adult supervisor), and walking-to-school day events
Allais et al. (46)	300 users of the transport system	3 groups, 2 intervention and 1 control group at metro stations	Filming of users with hidden cameras Measurement of stairway usage and frequency of usage	Controlled before-after	French National Nutrition and Health Program, 2011
Zhu et al. (47)	52 participants in the study and 36 in the post-test, 12 test control and 24 intervention	Two groups. The intervention was called stand up and move and a new adjustable workstation was provided compared to a control	ActivePAL3C to measure the activity, position and time MVPA level of activity	Two-arm non-randomized controlled trial	Part of the policy but does not express it,
Tarp et al. (48)	312 students from 10 public schools	2 groups from 14 schools in the municipality, 10 intervention and 4 control.	Blood, lipid and glucose profile measurements. Blood pressure and waist circumference measurements. Andersen's cardiovascular endurance test was performed to measure physical fitness with fast running. Activity measurements by self-recording and accelerometer level of physical activity from MPA- MVPA	Controlled before-after with control group	Aldersrelateret træning—Målrettet og forsvarlig træning af børn og unge. 2005, Copenhagen, Denmark: Team Danmark
D'Agostino et al. (49)	2,250 children aged 6–15 years	Program is 10 months of after-school activity	Sociodemographic data Measure of change in ethnic group segregation. BMI and fat folds according to CDC percentiles and systolic and diastolic blood pressure numbers. Aerobic capacity test with the 400-m run test	Before-after	CDC. Decrease in infant mortality and sudden infant death syndrome among Northwest American Indians CDC. A public health action plan to prevent heart disease and stroke
Nicosia and Datar (50)	749 children of military	Two groups, military transfer parents and non-transfer controls	Level of physical activity in minutes per week and perception of nutrition by intake Time and frequency of MPA-MVPA activity during the week Sites or scenarios available for the practice of activity with GIS system.	Before-after	U.S. Department of Defense. Overweight Children in the Military Health System. Washington,
Sun et al. (51)	Number of trips and change in the types of trips	An intervention group with a new subway line with 24 stations pre- and post-measurement	Questionnaire of the preferences and type of uses of transport, bus, bicycle, walking or car, how long and with what frequency	Before-after with control group	Part of the policy but does not express it,
Mölenberg et al. (52)	*n* = 1,841 ages 6 (2008–2012) and 10 (2012–2015). (*n* = 1,607) outside playground (*n* = 1,545). Sedentary behavior	Two intervention groups with 18 new spaces in economically depressed areas compared to a control	Distance from the houses and neighborhoods to sporting grounds. Use of spaces. Hours spent on activity in open environments during the week and at the weekend. Level of physical activity of the participants.	Prospective cohorts	World Health Organization, 2012. The Built Environment: Designing Communities to Promote Physical Activity in Children usa and Denmark
Kapinos et al. (53)	237 Students assigned to the dormitories	Pre- and post-intervention, the groups were worked according to distance from the dormitories to gyms and restaurants	Questionnaire, height and weight data, sociodemographic aspects, exercise and diet data with direct questions on frequencies and number of meals per day and nutritional level. Exercise and diet data with direct questions on frequency and number of meals per day and nutritional level, number of days and frequency of exercise and distance walked to use the areas.	Cohort pre and post with control group	U.S. Department of Health and Human Services. Healthy people 2010: With understanding and improving health and objectives for improving health, In: Services USDoHaH, Washington, DC
Sharma et al. (54)	329 women	Multi-component pre- and post-intervention program	Sociodemographic data. Data on environmental, psychological and behavioral aspects. Dietary behaviors related to frequency of consumption, type of food, physical activity in terms of intensity, duration and frequency. Physical activity in terms of intensity, duration and frequency; Psychosocial factors related to food security, attitudes and ways of eating.	Before-after	Early life-cycle approach in tackling obesity, while advocating for a holistic, systems-based per-spective in the formulation of policies and interventions
Madsen (55)	6,967,120 school district students	Pre- and post-California Fitness Program for children ages 5–7th and 9th	Physical fitness was assessed using the Fitness Gram. Body composition area, BMI and skinfolds or by electrical impedance.	Before-after	Policy Position Statement on Body Mass Index (BMI) Surveillance and Assessment in Schools. American Heart Association; 2008
Klakk et al. (56)	1,218 children	2 groups of 10 schools in the municipality, 6 intervention and 4 control.	Blood, lipid, and glucose profile measurements. Blood pressure and waist circumference measurements. Andersen's cardiovascular endurance test was performed to measure physical fitness with fast running. Activity measurements by self-recording and accelerometer for the level of physical activity from MPA-MVPA	Quasi-experimental.	Pryce R, Willeberg S, Falkentoft C, Meyhoff T: Aldersrelateret træning—Målrettet og forsvarlig træning af børn og unge. 2005, Copenhagen, Denmark: Team Danmark
Aittasalo et al. (57)	44 companies, 1,833 workers	11 companies. The presence of lanes or roads for cycling or walking and use by workers for active transport was determined compared to control	Questionnaires on the use of the bicycle or walking as a method of active transport Time of use in hours or minutes and number of times per week on the activity LPA/MPA/VPA level of physical activity	Randomized controlled trial	Ministry of Transport and Communications (Liikenne- ja viestintäministeriö). Program for Promoting Walking and Cycling (Kävelyn ja pyöräilyn edistämisohjelma). 2018
Stone et al. (58)	16 school districts, 1,027 children and parents	BEAT pre-and post-program, environmental project to encourage physical activity in school children in Ontario	Accelerometry School-day and school-time activity, measures of activity time in counts, frequency and intensity of MVPA	Cross-sectional before-after	Ontario Ministry of Education. Daily physical activity in schools: Guide for school boards
Azevedo et al. (59)	497 participants intervention *n* = 280; control 217	Two groups of 7 schools, intervention 5,280; control 2	Accelerometry to determine moderate to vigorous activity times, calculation of activity type according to counts Sedentary or active style according to level Anthropometric measurements of height weight, BMI by calculation and densitometry Aerobic capacity with the 20-m multistage running test for VO_2_ Cardiovascular response and self-efficacy of physical activity with the self-efficacy questionnaire for children and quality of life with the kids screen 27. Qualitative interview with teacher and student	Non-randomized controlled trial with qualitative study.	Part of the policy but does not express it
Heinen et al. (60)	4,279 users responded and were included in the study and 40% completed	The low-cost community bicycle habitat program in Australia, pre and post, with 2,000 community bicycles	Questionnaire psychological stages to cycling Change in transport activity pattern to cycling, use, frequency, use for recreation or use for commuting to work Calculated bicycle use time by self-reporting and determined bicycle use exposure by distance from work to home and other commuting sites	Cohorts	Brisbane City Council, 2016. *via*: https://www.brisbane.qld.gov.au/facilities-recreation/sports-leisure/cycling-brisbane
Sarmiento et al. (61)	4,925 park users	3 groups of parks with new playgrounds, old playgrounds and no playgrounds	SOPARC Types of areas and use Physical activity level MPA/MVPA	Cross-sectional Comparison pre/post with control group	Recreovía program in Bogotá, Colombia, Muevete Bogota
McGavock et al. (62)	176 users	Two intervention groups of two frozen waterways in winter	Number of counts of the use of the tracks in the groups by means of an infrared system Level of physical activity in users who attended the track with the use of pedometers on their waist MVPA and counts steps	Before-after with control group	Part of the policy but does not express it

Main methodological, LPA, light physical activity; MPA, moderate physical activity; MVPA, moderate-vigorous physical activity; VPA, vigorous physical activity; METS, metabolic equivalents; AC-R, Academia da Cidade; SOPARC, system to assess the practice of physical activity and recreation in parks and natural surroundings; IPAQ, physical activity questionnaire; RPARQ, recreation physical activity questionnaire; GPS, Global Positioning System; HEAL, Healthy Eating Active Living; PALMS, Physical activity location measurement system; ActivePAL3C, active https://journals.lww.com/epidem/Fulltext/2008/11001/Geospatial_Measurement_Analysis_Of_Physical.186.aspx Physical activity location measurement system; PACER, walk system measurement; BMI, body mass index; CDC, center disease control; VO_2_, volume Oxygen consumption; NIH, National institute of health; UTC, Universal Eligibility Criterion; TCT, Targeted Eligibility Criterion; SRTS, the federal Safe Routes to School; WSB, Walking School Bus.

### Measurements of physical activity in the studies

All included studies reported the outcome of PA with valid and reliable measurements as shown in Table 2. At the non-instrumental level, the IPAQ PA questionnaire was used in 2 studies, the SOPARC leisure or activity measurement system in parks in another 2 studies, and the RPARQ PA level measurement survey in recreation in one study (35–37, 40, 41). Fifteen studies provided levels of PA as light, moderate or vigorous (35–38, 40, 41, 47, 48, 50, 52, 54, 57–59, 62), and the hours/minutes of activity were reported in 19 studies (43, 50–52, 54, 57, 58, 62).

Objective measurement data of PA was measured with the following instruments including an accelerometer in 7 studies (35, 36, 40, 44, 48, 58, 59) a pedometer in 1 (62); and global or geographic location such as GPS, and ACTIVE-PAL-3C in 4 studies (36, 40, 47, 50). Finally, 8 studies reported other physiologic measures including anthropometrics, vascular resistance, METS, and blood chemistry such as lipid profile, cholesterol, triglycerides, and glycemia (38, 42–44, 48, 49, 55, 59).

### Effectiveness of the programs

The percentage of PA performed at the end of the intervention was examined in 10 studies (36, 38, 40, 41, 47, 48, 52, 58, 59, 61) as shown in Table 3. The level of PA in the majority of the populations was found to be light PA (LPA). For example, the reports in two of the largest studies, one conducted in Pernambuco Brazil on active cities (41) and another one recreo *via* in Colombia (61), found that 25.8 and 57.8% of the population performed LPA, respectively. In addition, the greatest effects of environmental interventions at the social level were in schools and workplaces. In schools, the percentage increase of PA was 2.3–6 points after a period of 12–16 weeks of the program (38). Regarding workplaces, the increase from moderate to vigorous activity was 19.3% in 1 week (47). In contrast, in two studies, one with an intervention of activity in the neighborhood in the cities (52) and the other examining bicycle commuting (36), no changes in the use of bicycle areas or boulevards were reported.

**Table 3:** Effectiveness of the programs.

	**Activity level**				**Sedentary time**	**Program impact**
**References**	**Counts/sample**	**Activity type**	**% Physical activity**	**Time physical activity (%)**		
Torres et al. (35)	1,533 participants 80% reported participating in the program for more than 3 months, 29% attending weekly and 43% monthly, 64% participating in classes AND (71%) Weekly class attendance	97% reported walking on the recreovía, cardiovascular (84%), walking or bicycling as Public Transportation (73%) and (18%), respectively.	-	↑ Number of minutes reported for leisure walking by 30 min compared to controls which has a decrease of 90 min. Recreational users *via* were more active on accelerometers relative to New users of vigorous 16 min at week ± 40, and at the weekend (79 min of MVPA ± 49) and at weekend 20 min MVPA ratio 225 start 305 finish	-	Positive evidence of the program at the district level with increased physical activity and inclusion of new users in higher levels of physical activity, this program being one of the ways to materialize the public policy.
Dill et al. (36)	↓ 307–240 and from 183 to 123 In second follow-up	_	↓ MV from 39.5 to 39.6%	↓ Total time on bicycle from 104 to 66 min and walking from 107 to 89	_	The active transportation program modifies habits in the population but does not favor an increase in activity during transportation time given the limitations presented in the study.
Prinss et al. (37)	414 participants	↑ in bicycle use 23.2% and 22.8% in each group	-	↑ minutes cycled between groups 85.4 (71.8) and 87.2 (74.9)	-	The effect of the commute program is important for increasing activity times on transportation as a public policy to encourage activity.
Gesell et al. (38)	82 children included	-	↑ Light total physical activity in the out-of-school intervention group (LMV) by 3.0 percentage points (*P* = 0.006), and 6 percentage points over 12-week study period and decrease in control group ↑ MVPA by a mean of 2.8 percentage points in each measurement period (*P* = 0.006), with a total increase of 5.6% points over the 12 weeks. The mean difference observed between the two groups of children who had data at week 12 was 10.8 (*P* = 0.001) percentage points in LMV and 13 percentage points in MVPA (*P* < 0.001)	.	-	Establishing community recreation centers that incorporate structured physical activity opportunities is associated with significant increases in physical activity during after-school activity time for public school children and could be a promising low-cost approach to improving children's health trajectories cost.
Barradas et al. (39)	1,533 participants.	-	-	↑ Total levels minute of leisure-time PA 158.1 (SD = 230.2) men 187.7 (SD = 245.3) women 145.8 (SD = 222.6) Moderate levels of leisure-time PA 81.9 (SD = 154.5) men 104.4 (SD = 176.8) women 72.5 (SD = 143, 2) Vigorous levels of leisure time PA 76.2 (SD = 160.2) men 83.2 (SD = 160.9) women 73.2 (SD = 159.8)	-	Participants reported elevated levels of both HRQoL and Life Satisfaction LS. Participants who reported higher LS scores also reported higher levels of leisure-time PA. No differences were found in differences between HRQoL scores and leisure-time PA. The second objective of the study was to differentiate levels of HRQoL and LS among Recreovía participants. Participants in Recreovía showed better indices of psychological wellbeing, highlighting the potential of the program to improve physical health.
Goodman et al. (40)	-	↑ 5.81–6.78% prevalence of cycling to work in 2011. Relative effect of 1.09 (95% CI: 1.07, 1.11). ↓ Prevalence of driving to work [−3.01 (−3.13, −2.88)]. 14 out of 18 cities ↑ higher cycling prevalence in 2011	Increased prevalence of walking to work [+1.71 (95% CI 1.62, 1.81)] percentage points lesser extent, of public transport use [+0.32 (0.24, 0.41)] percentage points	-	-	City-level interventions have potential health and environmental benefits, cycling is accompanied by decreased car commuting to work and increased commutation with lifestyle modification. The results indicate that city and cycling city initiatives have so far promoted cycling for healthy commuting and health equity, while also providing environmental benefits.
Simões et al. (41)	10,000 participants	**_**	The proportion of individuals that reached the LPA guidelines was 25.8%	For those who never participated and began their participation and to reach the levels (OR = 1.61; 95% CI 1.18; 2.20, < 6 months 1.83; 95% CI 1.17; 2.86, *p*-value = 0.0078) more than 6 months (OR = 5.06; 95% CI 3.34; 7.67, *p*-value b0.0001)	**_**	The community-based physical activity intervention had a positive impact on LTPA levels in the population, especially among women. Evaluation of complex programs such as AC-P is feasible, with the study design and flexibility to rapidly fund and implement the study.
Esdaile et al. (42)	720 children the total number of sessions ↑for children enrolled in groups with UEC (Mdn = 7, IQR = 4.25–9, Mean Rank = 387) than for children enrolled in groups with TEC (Mdn = 7, IQR = 3–9, Mean Rank = 352), *U* = 43,178.5, *p* = 0, 049 two-tailed	**_**	**_**	-	**_**	Program results suggest that families with overweight children are more likely to enroll in a healthy lifestyle program without weight criteria, in which marketing is aimed at improving healthy lifestyle behaviors, than in a weight management program with specific eligibility criteria. The program is also likely to have eligibility criteria and recruitment materials focused on healthy weight.
Kapinos and Yakusheva (43)	488 students	Dorm 7 houses one of the campus gyms and dorm 2 is only 0.13 miles from dorm 7. Despite exercising more frequently, only females assigned to dorm 2 weighed significantly less in the spring.		Although male students reported exercising more frequently on average, both males and females reported exercising less frequently during the first year compared to the year prior to entering college. Females in dorms 2 and 7 exercised more frequently during the first year.		
Hunt et al. (44)	26 children	PACER for walking ↑ median, but the change was not statistically significant (baseline = 11 laps, outcome = 14 laps, Δ = 3.00 laps, *p* = 0.26). On program break weekends, children accumulated 17.0 min less MVPA (72.4 min, SD = 45.5). And 13.5 min less AFMV (75.9 min, SD = 45.0).	**_**	89.4 min of AFMV (SD = 38.6) in the program. On days when the program ran but children did not attend, they accumulated 11.3 min less AFMV (78.1 min, SD = 38.0). During the week of the program break, children accumulated 10.0 fewer min of MVPA (79.4 min, SD = 37.3). Program attendance with MVPA was 45 min compared to 24 min for children when they did not attend the program or program break	**_**	This finding suggests that attendance at a structured summer program may mitigate BMI gain and loss of CRF, the impact of a structured program on weight gain and fitness loss, as well as obesogenic behaviors. Children maintained fitness, BMI, zBMI, and BMI percentile from the beginning to the end of the SLP.by helping children adopt healthier behaviors.
Lee et al. (45)	Out of 165 subjects 68 changed to active transport	41% active transport by bicycle or walking, 58.8% no change	**_**	**_**	_	The study notes that the shift from sedentary to active mode is associated with perceived environmental changes, such as shorter travel distance, improved safety conditions on the way to school, and greater availability of programs to support walking to school. This study offers some initial insights into additional factors, beyond the obvious distance factor, associated with mode shift.
Allais et al. (46)	205 individuals (49, 69, and 87 for the easy, health and control groups, respectively).	**_**	-	↑ Use of stairs at the beginning of the intervention in both the health and easy groups, with stronger effects for the latter but not maintained over time.	**_**	No differences between the treatment and control groups in the number of times individuals reported playing sports in a week. The stair use Advertisement program did not create a habit of stair use. At best, the effects of the PDPs lasted 2 weeks after the end of the intervention. As mentioned at the end of the Introduction, one effect of programs that encourage investment activities is to encourage the use of stairs.
Zhu et al. (47)	_	-	↑ 24.9–17.5 LPA and ↓ 6.6 to 6.5 MPA	_	↓ 337–281 sitting and ↑ 111–165 sedentary time	Natural experiment with high ecological validity with an intergroup design and a strong comparison group. The intervention group showed less prolonged standing at the workstation. The effect appears to have been sustained for 18 months, with concomitant improvements in cardio-metabolic and productivity outcomes.
Tarp et al. (48)	495 children Structured participation in leisure-time physical activity [odds ratio: 0.79 (0.46–1.36)], differed significantly between intervention and control	_	% MVPA/day [unstandardized beta: −0.17 (– 0.67 to 0.33)], nor mean counts/minute [unstandardized beta: −25 (−58 to 8)].	As for the blood chemistry variables by increase over time, the differences expressed in untransformed scales were −0.03 (−0.12 to 0.06) mmol/l, −0.08 (−0.24 to 0.08) and −0.10 (−0.33 to 0.14) for triglycerides, TC: HDL-c and HOMA-IR, respectively	On non-transformed scales, differences between intervention and control schools were −0.3 (−2.1 to 1.5) mmHg, −0.2 (−1.6 to 1.2) centimeters and −9 (−39 to 20) meters for systolic blood pressure, waist circumference and cardiorespiratory fitness, respectively.	Despite the effectiveness of the intervention over 2 years, tripling curricular physical activity from kindergarten to grade 6. did not result in a significant reduction in the number of children in the classroom or the number of clustered or individual biological risk factors between intervention and control schools, when assessed after 6.5 years.
D'Agostino et al. (49)	2,250 children			Girls who had decreased segregation showed greater improvement in all outcomes cardiovascular activities compared to boys Both non-Hispanic Afro and Hispanics who had decreased segregation	Non-Hispanic Afro showed greater improvements in skinfold thickness, SBPP, and running time, while Hispanics showed greater improvements in BMIP and DBPP 187–126 sg in 400-m run in cardiovascular health 140–104 sg in 400-m run	Worldwide, parks are accessible to the public and should be considered a valuable global resource in the effort to prevent childhood obesity and promote health equity. Effective global public health policy must address health inequalities through targeted prevention strategies and resource-based health equity. The United States suggests that increasing population physical education in public schools is a cost-effective method to reduce the burden of hypertension and reduce the burden of cardiovascular disease attributed to hypertension.
Nicosia and Datar (50)	829 children.		By type of PA, the association of interest was significant only for vigorous PA, but never for moderate PA. For vigorous PA, the coefficient coefficient of the interaction statistically significant (coefficient 12, 5, po0.05), those living outside the facility (coefficient 18.6, po0.05), and only for those who moved (coefficient 12.1, po0.05).	Who had moved recently from those who had not, the association of interest was positive and significant among those who had moved less recently (coefficient 21.7, po0.05), but not among those who had moved more recently. The coefficient was higher among less recent movers who consistently live away from home (coefficient 35.9, po0.01)	_	This study suggests that greater access to PA opportunities in neighborhoods may be an important avenue for increasing PA among adolescents. The focus on children in military families could raise concerns of generalizability. However, the majority of the sample did not meet recommended levels of PA, similar to the general population. The results might not be generalizable to younger children who rely on their parents for PA or to adults with stronger habits. The natural experiment addressed assignment to location in terms of facility and individual-level fixed effects but did not address unobservable facility variables and individual-level fixed effects over time.
Sun et al. (51)	↓ 5,436–1,770 participants	↓ % of time journeys for work and not walking bicycle and bus between 2 and 28% in each, and increase in metro, car and metro from 28 to 33%	**_**	**_**	_	Natural experiments are becoming an increasingly popular tool to help transportation and health researchers generate better evidence when real experiments are not possible. The results the context of a developing city provide new evidence of the impact of the new subway on modal commute and active travel, new urban trains or urban rail system does not necessarily encourage increased active travel or reduced car use. Finally, knowledge of urban and transportation planning can help design and develop complex natural experiments on transportation and health.
Mölenberg et al.(52)	171 children participated in the use of 600 m of new spaces	.	Having 600 m of space dedicated for PA % no change in outdoor play in children 6–10 years compared to control	Children aged 10 years played 40 min more and in families with low maternal education level the children played 96 min more during the week	Reducing the distance to 100 meters did not present effects in sedentary behavior or increase in activity	The introduction of spaces dedicated to PA can increase outdoor play time and change in sedentary behaviors for children from more socioeconomically disadvantaged families. 10-year-olds with a nearby PA space played 0.5 h/week more outdoors compared to children without dedicated PA spaces around the house. In the case of children from families with a lower maternal educational level, outdoor play was 1.5 h/week higher. These estimates are larger than those found in the experimental (natural) setting, suggesting that both selection and causal mechanisms may explain the relationship between access to play facilities and physical activity.
Kapinos et al. (53)	1,935 participants Differences in changes according to distance to gym in 5 h per week by proximity	No effect of Proximity to gym on BMI for females, those living within 0.39 miles of a campus gym more likely to exercise frequently (more than 5 h per week), females living 0.39 miles or farther away less likely to exercise frequently.		Proximity to a campus gym had no effect on exercise frequency for males. Males living more than 0.39 miles from the nearest campus gym had significantly lower BMI and those living closer were significantly less likely to exercise.		Exogenous changes in the physical activity environment may lead to changes in weight and related behaviors but we failed to provide clear and robust evidence for such a relationship. Understanding spatial effects is challenging, as simple linear distances may not capture the implicit cost of using nearby physical activity services.
Sharma et al. (54)	210 women	14% increase in the number of women who reported being able to walk at least 10 min 5+ days per week	-	Physical activity for a total of at least 30 min during the past 7 days 3+ days per week from 82 to 113 Walking at least 10 min in a row for the last 7 days from 97 to 125 15% increase in the number of women reporting themselves active for at least 30 min per day 3 or more days per week	–	Programs such as HEAL provide a framework for successfully initiating clinic-community linkages and demonstrate the initial feasibility and acceptability of their implementation. HEAL demonstrates the feasibility of implementing this framework at the clinic and community level, >95% fidelity in program implementation, and acceptability of program strategies. By integrating a primary prevention approach to childhood obesity into the healthcare system, HEAL aims to create a model for system-level approaches to childhood obesity prevention, beginning in pregnancy. The study demonstrated an increase in physical activity among HEAL participants before and after the intervention, each week the women participated in physical activity sessions.
Madsen (55)	6,967,120 students	_	_	Valid BMI data for 6,967,120 students, representing 72.7% of all 5th, 7th, and 9th graders for the years 2001–2008	_	Widespread use of BMI screening and reporting is encouraging, as it reflects the willingness of schools to devote resources to addressing the obesity problem. In addition, research could explore how this type of information could be used more widely with other stakeholders and in policy. In the meantime, schools are likely to reap greater benefits if resources are used o increase opportunities for physical activity and improve nutrition.
Klakk et al. (56)	1,218 (81%), 697 of 773 (90%) from intervention schools and 521 of 734 (71%) but with different measures control	The difference in changes between intervention and control for TC: HDL, WC and CRF was small and insignificant. CRF 896–967 mt int and 893–961 mt cont	Six physical education classes per week significantly changed children's composite CVD risk score in favor of children attending intervention schools.		Mandatory physical education intervention with six lessons per week in public schools may reduce cardiovascular risk factors in children. The effect size observed in this healthy pediatric cohort, with the largest effect in the subgroup with the poorest composite risk score, which encompasses children in need of prevention, underscores the potential for school-based intervention programs.
Aittasalo et al. (57)	↑ 646–1,013 cycling and 309 to 346 walking	↑ Commute to bicycle 36% and walking 11%	_	↑ Commute time from walking and cycling for transport	_	The present study uses a socio-ecological framework in promoting commutation in a way, which has not been used in previous studies. Environmental improvements were part of the city's traffic plans and social and behavioral strategies. In addition, the intervention included several types of workplaces and the feasibility of the protocol related to the social and behavioral strategies had been previously tested.
Stone et al. (58)	856 participants 16.6% participated in daily activity on 2 days, 17.9% on 3 days, and 16.1% on 4 days		19.3% of participants (*n* = 165) accumulated at least 1 sustained session (≥5 min) of MVPA during the school week. The proportion varied among the 16 participating schools (0–45%). Most children (74.5%) accumulated 1 session, while 18.2 and 3.7% accumulated 2 and 3 sessions, respectively; only 6 children (3.6% of the sample) accumulated 4 sessions	The overall intensity of their activity was activity was higher and they accumulated significantly more minutes of moderate to vigorous activity throughout the school days (MVPAWD) and during the school day period (MVPASD) TPAWD 422. 429 (124,245) to 460,778 (135,477) MWD 437. 5 (140.9) A 463.9 (166.4) MVPAWD 30.2 (13.8)A 34.1 (16.1) MVPASD 15.1 (7.3) 18.0 (8.8)	DPA frequency was positively associated with total physical activity 423,386 (126,369), mean counts and cumulative weekday MVPA minutes (*r* = 0.10–0.13, *p* < 0.01). 29.6 (13.5) DAYS	The objective of this paper was to assess whether the Ontario Ministry of Education's daily physical activity policy (DPA) is being effectively implemented in elementary schools. The results show that most schools do not meet the required frequency (5 days) or intensity (maintaining vigorous activity for at least 20 min) of the DPA policy. However, our work demonstrates that frequency and intensity of DPA is positively related to student health behaviors/outcomes. Although our design prevents us from determining cause and effect, a positive relationship between DPA and physical activity/health in children clearly exists. Longitudinal studies are needed to establish whether benefits in students when the policy is effectively implemented.
Azevedo et al. (59)	497 participants (intervention *n* = 280; control *n* = 217).	There was no statistical difference between intervention and control participants between follow-up adjusted means for self-efficacy for physical activity or aerobic fitness.	Percentage of light physical activity (mean difference = −2.3%, 95% CI = −4.5 to 0.2, *p* = 003 MVPA (min.d-1) Basal 52.2 ± 16.4 post 58.2 ± 16.0 diff −5.6 (−13.6 to 2.3)	Light physical activity (min.d-1) basal 205.6 ± 36.0 post 234.3 ± 36.4 diff = −28.7, (95% CI = −46.5 to −10.8, *p* = 0. 02), MV (min.d-1) Basal 52.2 ± 16.4 post 58.2 ± 16.0 diff −5.6 (−13.6 to 2.3). Total MV activity (counts min-1) basal 892.5 ± 187.2 post 993.0 ± 230.7 diff −100.5 (−193.3 to −7.6)	Sedentary time (min. d-1) BASAL 502.3 ± 66.5 (152) POST 512.7 ± 63.5 (32) percent sedentary time (mean difference = 3.3%, 95% CI = −0.7 to −5.9, *p* = 0.01)	Implementation of a dance mat exergaming scheme in public high schools was associated with an improvement weight, BMI, body fat percentage and some parameters of health-related quality of life, but not with aerobic capacity, self-efficacy for physical activity or school attendance.
Heinen et al. (60)	4,637 respondents	No statistically significant associations. between proximity to a bike share station and changes in time spent cycling		Reduction in total time spent cycling by 1.98 min per week. Average time spent 8.8% (*n* = 362) increased their total cycling time by 35 min or more in 1 week, 81.5% (*n* = 3,356) changed their total cycling time by < 35 min. 9.7% (*n* = 400) reduced total cycling time by 35 min.		Our results indicate that residential proximity to a bike share station was not significantly associated with a higher level of (intention to) use nor with a greater propensity to increase total time spent bicycling, perhaps due to the older cohort in our sample. Studies have indicated that older people are less likely to adjust their travel behavior compared to the younger age cohort.
Sarmiento et al. (61)	4,925 users Parks with existing recreational pathways *n* = 994 % 29.9 Parks implementing future recreational pathways *n* = 147% 29.8 Control parks *n* = 338% 33	Women aerobic (7.7%), walking (7.0%) and basketball (6.6%). less frequent swinging (0.6%) and running (0.5%), parks with existing Recreovía, aerobic 21.2%). parks with future Recreovía, the main activity skating (5.9%). control parks activity carried out basketball (11.4%). Men soccer (14.3%), basketball (10.1%) and standing (8.5%). least common jogging/running and stretching (0.6%),	Mild Parks with existing recreational trails *n* = 991% 57.8 Parks implementing future recreational trails = 106% 50.3 control parks *n* = 144% 44.7 Vigorous: Parks with existing recreational trails *n* = 287% 16.8 Parks implementing future recreational trails = 39% 18.5 control parks *n* = 35% 10.9	women parks with existing Recreovía moderate to vigorous physical activity (MVPA), compared to women observed in parks without Recreovía 75 vs. 61%; *p*-value < 0.00 Males more likely to engage in MVPA in parks without Recreovía vs. parks with Recreovía 71 vs. 65%, (*p*-value < 0.01) 1		Parks with Recreovía were more likely to be used by women and had a higher percentage of users compared to parks without the Recreovía program. The presence of the Recreovía program was also associated with higher levels of MVPA observed among women. Providing culturally appropriate PA and dance classes and dance classes in public parks on weekends could be a promising strategy to promote PA among women.
McGavock et al. (62)	↑ 405–1,813 and 2,449–4,516 in two follow-ups 4,195 steps in 39 min, 4,796 vs. 3,987 steps during the week	**_**	**_**	↑ MVPA in minutes (32 vs. 25 min) and accumulated 27 ± 18 min of MVPA	**_**	The creation of a trail on a frozen waterway resulted in a significant increase in visitors to a network of urban trails. The activity dose that users achieved while on the frozen waterway was within the range necessary for health benefits. Trail users reported significant health benefits associated with trail use. Frozen waterways are a novel population health intervention to support increased physical activity after the winter vacations.

Effectiveness of the programs, PA, physical activity; LPA, light physical activity; MPA, moderate physical activity; MVPA, moderate-vigorous physical activity; VPA, vigorous physical activity; METS, metabolic equivalents; MVPAWD, moderate-vigorous physical activity week day; MVPASD, moderate-vigorous physical activity school day; DPA, Diary physical activity; HRQoL, health related quality of life; TC, total cholesterol; HDL, high density Lipids; CRF, Cardiorespiratory function; CVD, Cardiovascular Disease; BMI, Body mass index; HEAL, Healthy Eating Active Living; PDPs, Point-of-decision prompts; PACER, walk system measurement.

The increase of PA as an outcome was reported in 14 records corresponding to 11 studies (35, 37, 40, 43–45, 51, 54, 57, 59, 60). In these studies, aerobic activities such as running, jogging, or walking were implemented. Also, the use of bicycles as an activity, commuting as a means of transportation, or as a method to access public areas was found (35, 37) with bicycling being the most effective as a means of transportation reported in 7 studies (35, 37, 40, 45, 51, 57, 60). One of these studies (40) reported an increase in the prevalence of bicycle use from 5.81 to 6.78%, with an Odds Ratio (OR) of bicycle use of 1.09 (95%CI: 1.07–1.11), and a decrease in the use of the vehicle with an OR of 3.01 (95%CI: 3.13 to −2.88). Three studies found an increase in school activities and walking, and also intra- or extracurricular PA with two of the studies in schools (44, 59) and one in parks (35). The study examining PA in parks found an increase of 97% in walking on the playground, 84% in cardiovascular activities, and 18% in cycling as transportation.

Changes in the time of PA were also examined of which 15 of 22 studies) (35–37, 39, 41, 43, 44, 46, 48–50, 52–62) demonstrated that the changes generated an increase or modification in minutes spent in PA, whether it was daily, weekly, and total PA for the population. Eight studies in the school area (43, 44, 48, 49, 55, 56, 58, 59) reported an increase of 89.4 min of weekly PA in students with increases in LPA from 422 to 460 min, vigorous PA from 30 to 40 min per week, and total PA from 187 to 230 min per week. Similarly, the studies of PA in parks and cities (35, 41, 50, 52) found an increase in the minutes of participation in recreational pathways, parks, or modified city areas, with an accumulated total time of 27 min of moderate-vigorous PA (MVPA). An improvement in the number of women actively participating in provided programs was also reported, with an increase in walking time that was >30 min with a variation of 82 (54, 61). In children, an increase in PA of 40 min was observed, and in children over 10 years of age or in children from economically deprived families, the increase in PA time ranged from 96 to 113 min (50, 52). The study of Brazilian cities (41) demonstrated a dose-response relationship where a stronger association with adherence to leisure time PA guidelines was found the more exposed the population was to the program and whether the exposure was current compared to a past exposure. In this same sense, commuting as a form of transportation increased the time spent cycling or walking as shown in Table 3.

Finally, three studies reported a change from sedentary to active lifestyle (47, 52, 59) with an increase in PA time and a decrease in sedentary time highlighted by a diminution in sitting time from 337 to 281 min in participants who were examined in the workplace, school children, and city programs.

### Risk of bias in the included studies

Natural experiments are very useful in public policy due to the fact that the population is assessed in their environment at the time that programs or policies are implemented (63, 64). At the same time, one weakness of natural experiments is the risk of bias. Table 4 illustrates the results of the risk of bias assessment of the studies included. As shown, the risk of bias differs among studies, but there is an implicit risk of bias in natural experiments in the pre-intervention, during intervention, and post-intervention periods.

**Table 4:** Risk of bias in the studies included.

**Author/ measurement**	**Pre-intervention**		**During intervention**	**Post-intervention**		
	**Confounding**	**Selection**	**Intervention measures**	**Interventions performed**	**Outcome measures**	**Reporting bias**
Torres et al. (35)	X Lack of control of variables	X Selection by recreation *via*	√	√	X Measurements in subsamples not the whole population	√
Dill et al. (36)	X Lack of confusion management	X Selection of participation in boulevard	√	X Losses to follow-up	X Measures vary among participants as there are losses to follow-up	√
Panter et al. (37)	√ Protocol	X Selection of participation	√	√	√	√
Gesell et al. (38)	√	X Broad inclusion criteria	√	√	√	√
Barradas et al. (39)	X Lack of control of variables	X Selection by recreation *via*	√	√	X Measurements in subsamples not the whole population	√
Goodman et al. (40)	√ Protocol	X Selection of participation	√	√	√	√
Simões et al. (41)	X Lack of control of variables	X Selection by participation in the cities	√	√	√	√
Esdaile et al. (42)	X Lack of control of confounding variables	X Selection is by entry into the Peach program	√	√	X Measures focus on BMI	√
Kapinos and Yakusheva (43)	X Lack of control of variables	X Selection by allocation of bedrooms	√	√	X Bias due to self-reporting measures or no direct measurement of anthropometric changes	√
Hunt et al. (44)	√	X Broad inclusion criteria in after-school program	√	√	X Measurements are not population-wide	√
Lee et al. (45)	X Lack of control of confounding variables	X Selection is by entry into the Peach program	√	√	X Retrospective measures	√
Allais et al. (46)	X Lack of control of confounding variables	X Selection is by use of subway stairs	√	X Loss to follow up.	√	√
Zhu et al. (47)	X Lack of control of confounding variables	√	√	X Loss to follow-up	√	X Non-uniform measurements in the two groups
Tarp et al. (48)	X Confusion present no nutritional information in the analysis	X Selection bias, although it establishes entry criteria	√	X Loss to follow-up and data.	√	√
D'Agostino et al. (49)	X Lack of control of confounding variables	X Selection by participation in the program although there are broad criteria	√	√	√	√
Nicosia and Datar (50)	X Confusion present due to program entry at military bases	√	√	√	X Risk of bias measured by self-reporting	√
Mölenberg et al. (52)	X Confusion because it is handled by participation in the new spaces of the city √	X Selection bias due to not having clear inclusion criteria	√	X Loss of sample of the subjects evaluated because they lived far from the selection area	X Not having GPS measurements that determines the distances of the children to the work areas, and memory bias in the parents could affect the measurement	√
Kapinos et al. (53)	X Lack of control of variables √√√	X Selection by allocation of bedrooms	√	√	X Bias by self-report measures or by not directly measuring anthropometric changes	√
Sharma et al. (54)	X Risk due to control of confounding variables	√	√	√	√	√
Madsen (55)	X Risk due to control of confounding variables X √√ √	X Selection by allocation fitness program	√	√	X Risk in measurement only imc measure is reported as indirect measure of activity	√
Klakk et al. (56)	√	X Selection bias, although it establishes entry criteria √	√	X Loss to follow-up and data	√	√
Aittasalo et al. (57)	X Attempt is made to control variables but missing √	X Selection by use of transportation	√	X Loss of sample of subjects evaluated	X Bias by self-report measures and loss in accelerometry measures	X Attempt is made to control variables but missing √
Stone et al. (58)	√ Protocol	X Convenience selection bias	√	√	X Loss of measured variables due to incomplete data	√
Azevedo et al. (59)	X Lack of control of variables	X Differences in baseline between schools	√	X Loss to follow-up and data	X Loss of measured variables due to incomplete data	√
Heinen et al. (60)	X Confusion present due to participation in the program.	X Selection due to participation in the bicycle programs	√	X Loss to follow-up	X Bias due to self-reporting measures and loss in measures	√
Sarmiento et al. (61)	X Lack of control of variables	X Selection by recreo *via*	√	√	√	√
McGavock et al. (62)	X Lack of control of variables	X Selection by frozen channel use	√	√	X Bias by measures of register of channel use	√

## Discussion and conclusions

This systematic review focused on environmental social strategies to increase PA. The results found multiple social programs worldwide were studied through natural experiments. Twenty-four experiments from 28 reports developed in different environments such as schools, workplaces, streets or cities, neighborhoods, and parks were reviewed and analyzed to determine the effectiveness of promoting PA in populations. Of the included studies, 12 were carried out in external environments such as parks, cities, neighborhoods, or crosswalks, and the other 12 were carried out indoors or outdoors such as in schools and companies. The experiments provided innovative proposals for social programs that seek to increase PA and promote healthy lifestyles related to public policies developed in the countries in which they were generated.

Worldwide, environmental modification programs from the social perspective have gained relevance for the implementation of policy-based programs in countries whose impact has been evaluated through natural experiments (26). Natural experiments have strengths and weaknesses inherent to their methodological design and the scope of their conclusions. These studies have a higher risk of bias given population selection and confounding in the management of variables. But it is important to note that, although they have these central problems, they allow the analysis of community or environmental interventions in large populations and groups. In our systematic review, natural experiments were of vital importance given the prospects of working on PA from a population standpoint and reducing chronic non-communicable diseases as established by the WHO (65).

The use of natural experiments and their impact on the modification of public health problems like our study have been presented in three key studies. One of the largest studies was reported by the WHO in a different area with three large projects. The first was from Austria about the regulation of trans fatty acids to prevent mortality from all cardiovascular causes and coronary heart disease (66). The second was from Russia on the effects of tobacco control policy to prevent cardiovascular disease (67) and finally, a study from Romania on the increase in tobacco taxes (68). These three experiments from the WHO European project of natural experiments raise the strengths of their use in implementing public policies but their methodological weaknesses as well.

Another important factor to consider is the manner in which environmental modification and active transportation is related to health equity (69). A previous review included 28 studies carried out in adult and child populations. In contrast to our study, they included prospective, longitudinal, cross-sectional, repeated measures studies, and a natural experiment. Although the types of studies were different, all programs were focused on promoting PA through walking, bicycling, park-based programs, neighborhood modification, and even environmental recreation activities. Another difference was the list of risk of bias evaluation in which the instrument of evaluation of public policies in health practices of the Canadian Association for observational studies was used, but although the list was different, the evaluation was similar, finding weaknesses in the studies methodology but with the advantage in the description of the effectiveness of the promotion of PA. The previous research measured the activity reported ranging from the use of types of transportation to specific measures of activity in metabolic expenditure in METS or level of PA from mild to moderate to vigorous. Within the impact reports, increases in the number of users, metabolic work, or the level of moderate or vigorous activity were found to have a greater impact in school and adult physical activity programs, followed by those of parks or playgrounds modifications and those of urban renewal with the implementation of programs in the scenarios similar to our review.

In this same line, but in systematic reviews in different levels of evidence related to public policies and environmental modifications is an integrative review of systematic reviews and meta-analysis of urban modification and promotion of PA in Latin America. The results were reported in 14 articles and included 8 systematic reviews with studies of different levels from cohorts, cross-sectional, experimental, cases, and controls among others (70). The studies were developed especially in Australia, the United States, and England. The findings showed that programs which were proposed in the environment such as the development of bike paths or recreational spaces, transportation, and commute to active transportation increased the PA. Within the programs found there was evidence of an improvement in the levels of activity within a range of 8–33 min of walking per day with an increase in activity similar to that found in our results. In addition, the study found that the development of outdoor spaces that created scenarios in the population for the practice of the activity and the use of active transportation such as bicycles, walking at school and work level improved PA. The results of the study also suggest that the level of activity could rise by maximizing the use of physical spaces by satellite geo-referencing in neighborhoods and cities to increase activity and shows the relevance of developing public policies related to PA.

In the area of environmental programs focused on active transportation, there were two reviews, one systematic and the other synthesis of evidence from systematic reviews. The first was based on interventions to increase cycling (71) and the second was on urban environmental interventions to increase PA (72). In the first report with 12 studies, 2 clinical trials and 10 pre- and post-intervention of individual, group, and environmental interventions with outcomes to promote active transportation found that the implementation of programs focused on the individual or environmental infrastructure increases the level of transportation trips from 7 to 12%, with an OR of 7.8 in participants who rode a bicycle more than 2 km. Also an increment of 27.5% during the use of cyclists who use active transportation in the last 5 months, similar to what was reported in our study with the increase in time and number of trips. Similarly, an increase of 47.5% in the number of cyclists was found in a program in New Zealand where a bridge was constructed and not only improved PA levels, but also increased health status (71). Secondly, eight systematic reviews all of which were focused on the impact of urban interventions on PA demonstrated an increase in activities such as walking, cycling, switching from bus transportation to walking, or the use of bike lanes for the control of chronic non-communicable diseases (70).

Related to the topic of environmental modification, a systematic review but in different levels of evidence ranging from controlled trials to cross-sectional studies in the school setting, focused on in-school programs as in our study. The review of the effects of classroom-based programs on PA outcomes and academic performance (73) included 39 studies that examined the effect of activity programs in school settings. As in this review, there were programs to increase activity in the classroom for children and adolescents and included active rest periods based on aerobic activity to achieve the movement of students in the class to extra-classroom programs focused on sports or with additional equipment and implements to increase the level of activity. It is also noteworthy that the time and activity were variable among the programs ranging from 4-min of daily vigorous-level classroom activities to 20 min of moderate PA twice a week. Also, programs focused on the curriculum, such as the Ontario Natural Experiment have been studied in which in mathematics, language, science, or social studies classes incorporated cognitive academic skills with PA goals. In this paper three studies, two experimental cluster studies and one quasi-experimental study, were meta-analyzed to determine the effect of the program on PA, finding 95% heterogeneity with a non-significant effect of 0.40 CI −0.15 to 0.95.

About the applicability of these results in our review is important to consider since most of the studies come from high-income countries, and little information exists on middle- and low-income countries, likely because the urban modifications in-built environments is determined by the use of the land, density, and urbanization. This is important because economic and educational aspects influence the type of environmental interventions implemented and the possibility of changing behaviors in the population. The evidence shows some favorable results related to the implementation but stronger evidence is needed to determine the changing in behaviors (72, 74).

In conclusion the 24 reviewed studies suggest innovative proposals for social programs that seek to increase PA and promote healthy lifestyles related to public activity policies developed in the countries in which they were generated. Environmental social programs can positively impact PA levels among children and adults. It is important to highlight that these documents presented and this research reflect the importance of implementing public policies aimed at promoting PA from an environmental perspective. Structural modifications and the creation of social programs from socioecological perspectives allow the establishment of other perspectives of approaching PA that not only focus on the individual but also how changes in the environment facilitate the implementation of plans, programs, and public policies for PA. It appears important that a central mission of a country is to implement policies to promote PA with a comprehensive vision centered on the populations within a country.

## Strengths and limitations

This systematic review based on natural experiments has several advantages including (1) the examination of large populations in natural settings providing an understanding of the effect programs and modifications to existing program may promote PA and (2) examining the implementation and measurement of public policies and programs established in the studies. A risk of bias is implicit in natural experiments and may introduce bias in the selection, measurement, and reporting of results. Nonetheless, natural experiments are an important type of study for decision-making in public health and especially in assessment of PA in environmental interventions.

## Data availability statement

The original contributions presented in the study are included in the article/Supplementary material, further inquiries can be directed to the corresponding author.

## Author contributions

Overall content as guarantor: EH. Study concept and design: EH and PS. Screening titles and abstracts: EH, PS, and EC. Search and extracted the evidence: EH and EC. Writing and revising the manuscript for important intellectual content and approved the final manuscript: EH, EC, LC, and PS. All authors contributed to the article and approved the submitted version.
