# Supplementary material for: Impact of environmental interventions based on social programs on physical activity levels: A systematic review
Source: Front Public Health. 2023 Mar 23;11:1095146. doi: 10.3389/fpubh.2023.1095146 (PMC10078830; doi:10.3389/fpubh.2023.1095146)
Supplement: Supplementary file 1 [file Data_Sheet_1.PDF]

Estrategias de busqueda RS

PUBMED

#1 "Physical Fitness"[Mesh]

#2 "Exercise"[Mesh]

#3 "Physical Exertion"[Mesh]

4 "Sports"[Mesh]

5 "Walking"[Mesh]

6 “physical activity”

7 Bicycling

8 "activity habits"

9 cycleway

10 park

11 healthy park

12 active transport to school

13 built environment

14 Active travel

15 Infrastructure

#16 Natural experiment

((((((((((((Physical Fitness) OR physical activity) OR physical exercise) OR exercises) OR Physical Exertion) OR Walking) OR Bicycling) OR "activity habits") OR cycleway) OR park) OR active transport to school) OR built environment) OR Active travel) OR Infrastructure

((((((((((("physical fitness"[All Fields]) OR (Exercise)) OR (Physical Exertion)) OR (Sports)) OR (Walking)) OR (physical activity)) OR (Bicycling)) OR (activity habits)) OR (cycleway)) OR ("park"[All Fields])) OR (healthy park)) OR (active transport to school)) OR ((((((citizen science)) OR (community engagement)) OR (architecture)) OR (built environment)) OR (Active travel)) OR (Infrastructure))

(((((((("Health Promotion"[Mesh]) OR "Health Education"[Mesh]) OR "Patient Education as Topic"[Mesh]) OR "Primary Prevention"[Mesh]) OR "Primary Health Care"[Mesh]) OR "Public Policy"[Mesh]) OR "Program") OR “education program”) OR “community education”) OR “life styles”

("natural"[All Fields] OR "naturally"[All Fields] OR "naturals"[All Fields] OR "nature"[MeSH Terms] OR "nature"[All Fields] OR "nature s"[All Fields] OR "natures"[All Fields]) AND ("experiment"[All Fields] OR "experiment s"[All Fields] OR "experiments"[All Fields])
